# *o*-Aminoazotoluene, 7,12-dimethylbenz[*a*]anthracene, and *N*-ethyl-*N*-nitrosourea, which are mutagenic but not carcinogenic in the colon, rapidly induce colonic tumors in mice with dextran sulfate sodium-induced colitis

**DOI:** 10.1186/s41021-022-00240-7

**Published:** 2022-03-29

**Authors:** Atsushi Hakura, Naoki Koyama, Yuki Seki, Jiro Sonoda, Shoji Asakura

**Affiliations:** 1 Global Drug Safety, Eisai Co., Ltd., 5-1-3 Tokodai, Tsukuba, Ibaraki 300-2635 Japan; 2 Global Drug Safety (present affiliation, Advanced Data Assurance), Eisai Co., Ltd., 5-1-3 Tokodai, Tsukuba, Ibaraki 300-2635 Japan

**Keywords:** Colon, Cancer, Dextran sulfate sodium, Inflammation, *o*-Aminoazotoluene, 7,12-Dimethylbenz[*a*]anthracene, *N*-Ethyl-*N*-nitrosourea, Mutagenic non-carcinogen

## Abstract

**Background:**

Several rodent models with chemically induced colon cancer have been developed. Among these models, dextran sulfate sodium (DSS), a colitis inducer, combined with azoxymethane as a colon mutagenic carcinogen, is commonly used. We previously reported that although benzo [*a*] pyrene (BP) is mutagenic but not carcinogenic in the colon, it rapidly develops colon tumors at a high incidence/multiplicity after treatment with DSS. In the present study, we examined whether other colon-mutagenic non-carcinogens (CMNCs) induced colon tumors after treatment with DSS.

**Results:**

*o*-Aminoazotoluene, 7,12-dimethylbenz[*a*]anthracene, and *N*-ethyl-*N*-nitrosourea were selected as CMNCs. Male CD2F1 mice were orally administered CMNC for 5 consecutive days. After a 9-day dose-free period, mice were treated with 4% DSS in drinking water for 1 week. Three months after DSS treatment, colon samples were collected for histopathology and β-catenin immunohistochemistry analyses. All CMNCs in combination with DSS induced colonic adenocarcinomas at a high incidence/multiplicity in the distal and middle parts of the colon, coinciding with the location of colitis. Unlike in normal cells where β-catenin is exclusively located on the cell membrane, in adenocarcinoma cells, it was translocated to both the nucleus and cytoplasm or only to cytoplasm. The translocation of β-catenin is closely associated with colon carcinogenesis in rodents and humans. No colonic tumors or dysplastic lesions were found after exposure to either CMNC or DSS alone.

**Conclusion:**

We provided further evidence clearly showing that CMNCs can rapidly induce colonic tumors in mice with DSS-induced colitis, even if they are not colonic carcinogens.

## Introduction

Colorectal cancer is one of the most common human cancers in the Western world [1]. Rodent colon cancer models have been developed to understand the mechanisms underlying colon carcinogenesis and to investigate potential chemotherapy or chemoprevention regimens. These models include carcinogen-induced, genetically modified, and transplant models [1–3]. Carcinogen-induced models are highly reproducible and can be readily tested on non-genetically modified animals with different genetic backgrounds. In addition, the processes involved in the pathogenesis recapitulate human colon cancer, particularly, the early stages of this disease [4–6].

Among carcinogen-induced models, some models using a colon mutagenic carcinogen in combination with dextran sulfate sodium (DSS), a colitis inducer, are often used [4]. Azoxymethane (AOM) [4, 7], 1,2-dimethylhydrazine (DMH) [8, 9] and 2-amino-1-methyl-6-phenylimidazo[4,5-*b*]pyridine (PhIP) [10–12] are used as colon mutagenic carcinogens (referred to as CMCs) to initiate carcinogenesis. These CMCs can induce colonic cancer by themselves [13, 14]; however, colonic tumors are induced at varying incidences (0–100%), and it takes 3 months or longer to induce cancer, depending on the species/strains or dosing regimens [15, 16]. For these reasons, DSS is used, in combination, to cause colitis in the colon (well documented as a DSS-induced colitis model), thereby accelerating the development and progression of colonic tumors. DSS is not mutagenic in bacteria [17] and in murine mutagenicity tests [18] but is weakly carcinogenic in the rodent colon [19, 20], indicating that DSS potently promotes colonic carcinogenesis. However, even in such “short-term” colon cancer models, the induction of cancer generally takes approximately 10–20 weeks [4–6].

We previously showed that benzo[*a*]pyrene (BP), a colonic mutagen [21, 22], induced colonic cancer at a high incidence/multiplicity in a shorter or comparable period, required for cancer induction in models using CMC and DSS after the treatment of mice with DSS, despite it being a non-colonic carcinogen [23–25]. There is no evidence showing that CMNCs (colon-mutagenic non-carcinogens), except for BP, clearly induce the formation of colonic tumors at a high multiplicity/incidence in the DSS-induced colitis model. The purpose of the present study was to examine whether other CMNCs also act as initiators for carcinogenesis in a DSS-induced colitis model.

*o*-Aminoazotoluene (AAT) induces mutations in the colon, liver, kidney, and urinary bladder [13, 26, 27]; only hepatocellular adenoma/carcinoma and hemangioendothelioma in the lung have been generated in mice [14, 28]. 7,12-Dimethylbenz[*a*] anthracene (DMBA) induces mutations in the colon, bone marrow, liver, skin, and thymus in mice [13, 29]; murine tumors were generated in the vascular and nervous systems, skin, and malignant lymphoma tissues [30, 31]. *N*-Ethyl-*N*-nitrosourea (ENU) induces mutations in numerous organs of mice, such as the colon, small intestine, bone marrow, mammary gland, and liver [13, 32, 33]. Multiple tissues are known to be the site of tumor induction in mice, including the liver, Harderian glands, stomach, ovaries, lymphoreticular system, kidneys, mammary gland, uterus, nervous system, and lungs [34, 35]. For these three chemicals, the colon is not recognized as a target organ for murine carcinogenesis.

In this study, we showed that all these three CMNCs induced colonic cancer in the presence of colitis. In addition, we performed the immunohistochemical analysis of tumors with β-catenin, given that the nuclear accumulation of β-catenin is well documented to be closely associated with colon carcinogenesis in rodents and humans [4–6].

## Materials and methods

### Chemicals

*o*-Aminoazotoluene (AAT, CAS No. 97–56-3, purity > 97.0%) was purchased from Tokyo Chemical Industry Co., Ltd. (Tokyo, Japan). 7,12-Dimethylbenz[*a*]anthracene (DMBA, CAS No. 57–97-6, purity > 95%) and *N*-ethyl-*N*-nitrosourea (ENU, CAS No. 759–73-9, content: 43.2% in water with 1.7% acetic acid to prevent decomposition) were obtained from Sigma-Aldrich Co. LLC (St. Louis, MO, USA). Dextran sulfate sodium (DSS, CAS No. 9011-18-1, molecular weight: 36,000–50,000) was purchased from MP Biochemicals, LLC (Aurora, OH, USA).

For administration, ENU was dissolved in water for injection at a concentration of 1.1 mg/mL, and AAT and DMBA were dissolved in salad oil (Nisshin Oillio Group, Ltd., Tokyo, Japan) at concentrations of 12.5 and 2.5 mg/mL, respectively. DSS was dissolved in water at a concentration of 40 mg/mL (4%).

### Animals

Male Crj: CD2F1 (BALB/c × DBA/2) mice were obtained from Charles River Japan, Inc., Tokyo. All mice were housed in metal cages (one mouse per cage) and were fed a basal diet (Oriental CRF-1, Oriental Yeast Co., Tokyo, Japan) and tap water ad libitum, under controlled conditions of temperature (23 ± 3 °C), humidity (55 ± 20%), and light (12-h light/12-h dark cycle). They were quarantined and acclimatized for 1 week. The animals were assigned by stratified randomization to the two groups according to their body weights; mice from both the DSS- and CMNC/DSS-groups and both the non-treatment and CMNC groups were assigned to the heavy and light groups, respectively, to minimize the mortality of light mice, given that they are likely to be more sensitive to DSS-induced colitis than heavy mice.

### Experimental procedures

**Fig. 1:**
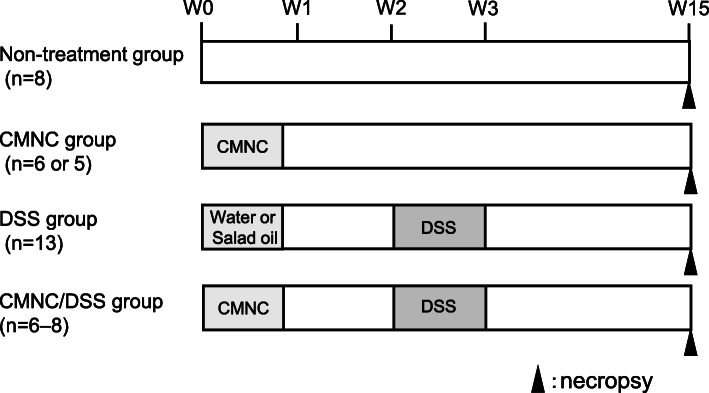
Outline of the protocol for the experiment for histopathology and immunohistochemistry of the murine colon. The experiment consisted of Non-treatment-, CMNC-, DSS-, and CMNC/DSS-groups. Male CD2F_1_ mice were treated with each CMNC at an oral dose of either 125 (AAT), 25 (DMBA), or 11 (ENU) mg/kg/day for 5 consecutive days, and starting 10 days after the last dose, mice were given 4% DSS in drinking water for 7 days. All surviving mice were necropsied 12 weeks after the last treatment with DSS. The number of surviving animals is shown in parentheses. Salad oil was used as the vehicle for AAT and DMBA, and water for injection was used as the vehicle for ENU

Figure 1 shows the outline of the protocol for the experiment. The number of mice, established upon initiation of the experiment, were as follows: eight for the non-treatment group, six for each CMNC group, 16 (eight for water for injection and eight for salad oil, vehicles with which CMNCs were dissolved) for the DSS group, and eight for each CMNC/DSS-group. Three of 16 mice in the DSS-group and one of seven mice in the DMBA/DSS-group died 4 or 5 days after the last DSS treatment, due to severe colitis, resulting in 13 surviving mice in the DSS group and 6 surviving mice in the DMBA/DSS-group. The numbers of surviving mice per group are shown in parentheses in Fig. 1 and Table 1.

**Table 1:** Incidence of colonic dysplastic foci and tumors induced by a CMNC in combination with DSS

Treatment	Dysplasia	Adenoma	Adenocarcinoma	Tumor^*a*^
Non-treatment	0% (0/8)	0% (0/8)	0% (0/8)	0% (0/8)
Vehicle^*b*^ + 4% DSS	0% (0/13)	0% (0/13)	0% (0/13)	0% (0/13)
AAT (125 mg/kg/day)	0% (0/6)	0% (0/6)	0% (0/6)	0% (0/6)
AAT (125 mg/kg/day) + 4% DSS	75% (6/8)***	100% (8/8)***	100% (8/8)***	100% (8/8)***
DMBA (25 mg/kg/day)	0% (0/5)	0% (0/5)	0% (0/5)	0% (0/5)
DMBA (25 mg/kg/day) + 4% DSS	50% (3/6)**	50% (3/6)**	50% (3/6)**	83% (5/6)***
ENU (11 mg/kg/day)	0% (0/6)	0% (0/6)	0% (0/6)	0% (0/6)
ENU (11 mg/kg/day) + 4% DSS	100% (7/7)***	100% (7/7)***	100% (7/7)***	100% (7/7)***

The numbers in parentheses indicate the number of mice with dysplasias, adenomas, or adenocarcinomas per the number of mice

CMNC; colon-mutagenic non-carcinogen

^*a*^: adenoma + adenocarcinoma

^*b*^: water for injection for 7 mice and salad oil for 6 mice

^**, ***^; significantly different from the DSS alone group at *P* < 0.05, 0.01 in Fisher’s exact probability test

For each CMNC/DSS-group, mice (7-week old) were orally (by gavage) treated with each CMNC for 5 consecutive days, and starting 10 days after the last dose, mice were administered 4% DSS in drinking water for 7 days. Twelve weeks later, the mice were necropsied under anesthesia to collect the colorectum from the cecocolic junction to anus for histopathology and immunohistochemistry.

The in-life phase of the experiment was performed at Sunplanet Co., Ltd. of the Eisai Co., Ltd. group, and the protocol was approved by the Institutional Animal Care and Use Committee and carried out according to the Sunplanet animal experimentation regulations.

### Tissue collection and histopathology

During necropsy, the large intestine was immediately excised, flushed with saline, infused with 10% neutral buffered formalin, cut open longitudinally along the antimesenteric border, and grossly observed. Thereafter, the tissues were stored in 10% neutral buffered formalin, cut into four parts of equal length from the proximal to distal ends, processed, and embedded in paraffin. Each colon sample was embedded to expose both longitudinally cleaved edges. All longitudinal sections were stained with hematoxylin and eosin (H&E) and histopathologically observed. The lesions of dysplasia, adenoma, and adenocarcinoma were classified according to the criteria that were described in detail in our previous report [24], which were originally reported by Riddell et al. [36]*,* Pascal [37] and Ward [38]. Dysplasia or dysplastic foci were characterized by irregular branching, distorted architecture with cellular and nuclear pleomorphism, nuclear enlargement and hyperchromatism, and paucity of goblet cells.

### Immunohistochemistry

Paraffin-embedded sections of mouse colons with adenocarcinomas from each CMNC/DSS group were subjected to immunohistochemical staining of β-catenin. Immunohistochemical staining was performed according to the procedure described in our previous report [24]. Monoclonal mouse anti-mouse β-catenin (clone 14, BD Transduction Laboratories, Lexington, KY, USA) was used at a concentration of 1/1000. After antigen retrieval, the Envision™ + Dual Link System or a streptavidin biotin-peroxidase complex method (DAKO, Glostrup, Denmark) was used to examine their expression and localization. These sections were counterstained with Mayer’s hematoxylin solution for microscopic examination.

### Dose setting for treatment with CMNCs

The doses of AAT (125 mg/kg/day), DMBA (25 mg/kg/day), and ENU (11 mg/kg/day) tested in this study were determined based on our preliminary study (data not shown) or the studies to measure the mutant frequency in mice; for AAT, a single *i.p.* dose at 300 mg/kg, corresponding to 40% of LD_50_ (median lethal dose) [26, 27], for DMBA, a single *i.p.* dose at 20 mg/kg [29] and for ENU, five *i.p.* doses at 22.2 mg/kg/day (once a week for 5 weeks) [39].

### Statistical analysis

The incidences and multiplicities were compared using Fisher’s exact probability test and Welch’s *t-*test for paired samples.

## Results

### Clinical findings

In DSS-treated mice, with or without CMNC, bloody and/or soft stools were observed for 1 or 2 days before the last DSS treatment. Stool changes resolved by 5 days after DSS treatment cessation and were attributed to colitis. In the CMNC/DSS groups, three mice that developed tumors showed bloody stools from 9 weeks after the last DSS treatment.

### Gross findings and histopathology

**Fig. 2:**
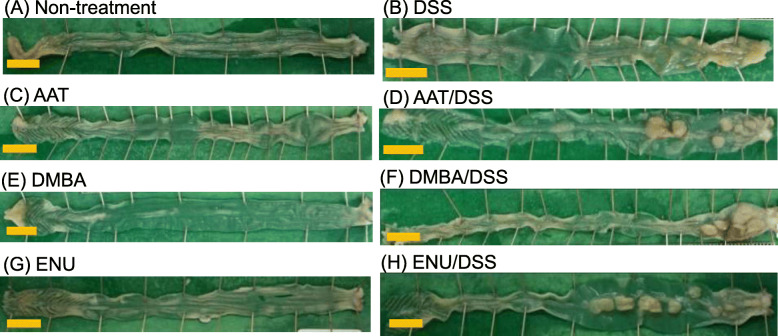
Macroscopic view of the colons of mice from Non-treatment- (**A**), DSS- (**B**), AAT- (**C**), AAT/DSS- (**D**), DMBA- (**E**), DMBA/DSS- (**F**), ENU- (**G**) and ENU/DSS- (**H**) groups. Bar: 1 cm

**Fig. 3:**
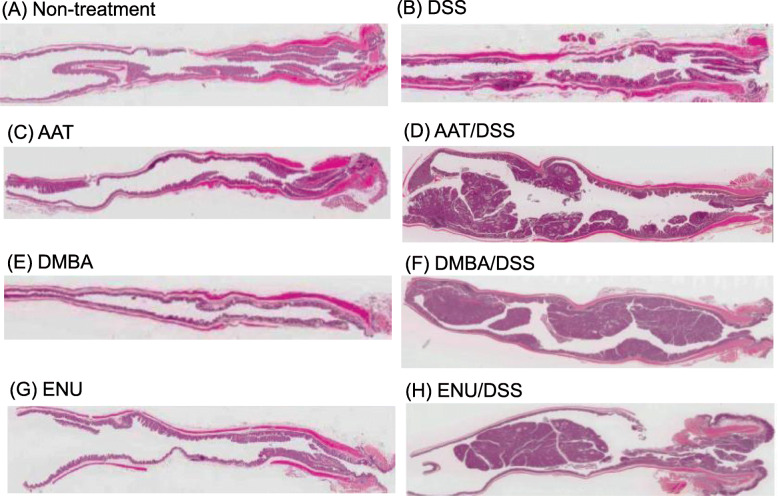
Histopathology of the colons of mice from Non-treatment- (**A**), DSS- (**B**), AAT- (**C**), AAT/DSS- (**D**), DMBA- (**E**), DMBA/DSS- (**F**), ENU- (**G**) and ENU/DSS- (**H**) groups. H& E stain

**Fig. 4:**
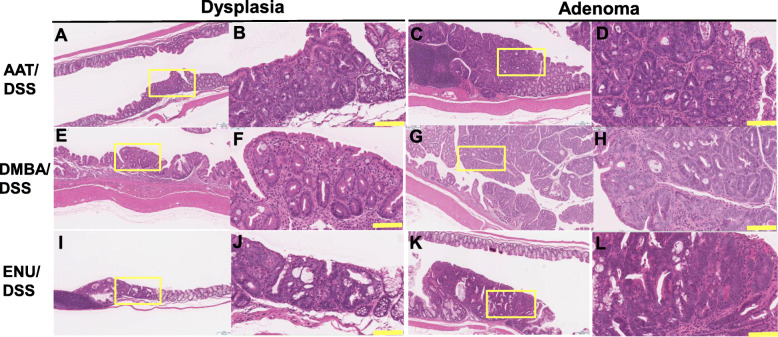
Histopathology results of H&E staining of colonic dysplasia (**A**, **B**, **E**, **F**, **I**, and **J**) and adenoma (**C**, **D**, **G**, **H**, **K**, and **L**) induced by AAT/DSS, DMBA/DSS or ENU/DSS. Figs. **B**, **D**, **F**, **H**, **J**, and **L** are enlarged magnifications of those in **A**, **C**, **E**, **G**, **I**, and **K**, respectively (the areas surrounded by the yellow frames). Yellow bar: 100 μm

**Fig. 5:**
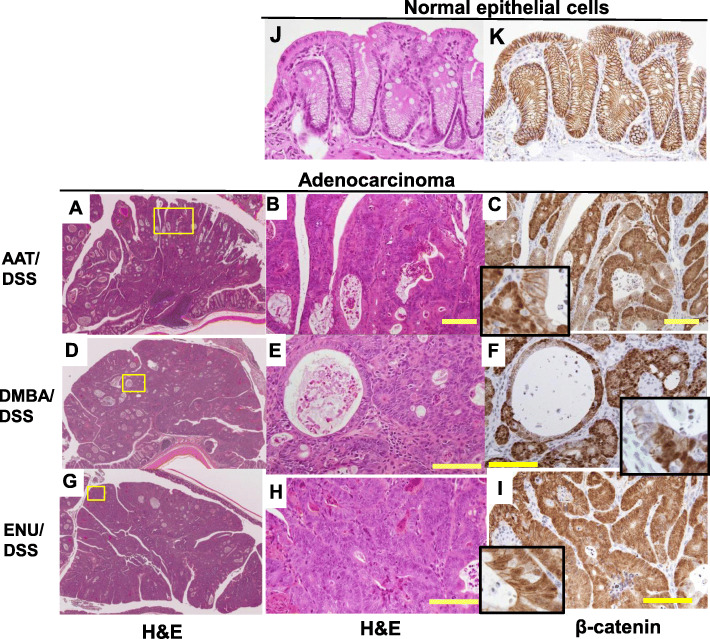
Histopathology results of H&E staining (**A**, **B**, **D**, **E**, **G** and **H**) and β-catenin-immunostaining (**C**, **F**, and **I**) of colonic adenocarcinomas induced by AAT/DSS, DMBA/DSS or ENU/DSS. Figs. **B**, **E** and **H** are enlarged magnifications of those in **A**, **D** and **G**, respectively (the areas surrounded by the yellow frames). Immunohistochemical analysis of β-catenin was performed on serial sections used for H & E staining. The inserts are figures in enlarged magnification. Figs. **J** and **K** show the results of H & E staining and β-catenin-immunostaining, respectively, of normal colonic epithelial cells. Yellow bar: 100 μm

The gross findings are shown in Fig. 2, and histopathological findings are shown in Figs. 3, 4 and 5. In the samples from the mice in each CMNC/DSS-group, there were multiple masses in the middle to distal parts of the colon at necropsy (12 weeks after completion of DSS treatment). Histopathological examination revealed dysplastic foci, adenomas, and adenocarcinomas in the distal and middle parts of the colon. Neoplasms were nodular, papillary, or polypoid masses composed of tubular and papillary proliferation of epithelium that protruded into the intestinal lumen, extended into the lamina propria, and compressed the adjacent mucosa. Tumors induced by these three CMNC/DSS were tubular adenomas or well- to moderately differentiated adenocarcinomas. No submucosal invasion or metastasis were observed. Pre-neoplastic and neoplastic lesions were not observed in the colon in any CMNC-, DSS-, or non-treatment-group.

In the CMNC/DSS- and DSS-groups, the large intestine was shorter in length, and the intestinal wall showed irregular thickening. Histopathologically, colitis with or without erosion or ulceration was noted in the distal and middle parts of the colon. In animals with colitis, there was occasional accumulation of foamy macrophages in the lamina propria.

### Incidence and multiplicity of dysplastic foci, adenomas, and adenocarcinomas

Table 1 shows the incidences of dysplastic foci, adenomas, and adenocarcinomas observed in the colon, and Table 2 shows their multiplicity (= the number of tumors/mouse). All CMNCs rapidly produced neoplastic lesions after combined treatment with DSS under the experimental conditions employed in this study. The combined treatments of ENU (11 mg/kg/day)/4% DSS and AAT (125 mg/kg/day)/4% DSS showed tumorigenicity in 100% mice, and the multiplicities are 8.6 ± 1.1 and 7.9 ± 2.0, respectively. The combination of DMBA (25 mg/kg/day)/4% DSS induced the formation of tumors in 83% of mice with a multiplicity of 3.0 ± 1.9.

**Table 2:** Multiplicity of colonic dysplastic foci and tumors induced by a CMNC in combination with DSS

Treatment	Dysplasia	Adenoma	Adenocarcinoma	Tumor^*a*^
Non-treatment	0 ± 0	0 ± 0	0 ± 0	0 ± 0
Vehicle^*b*^ + 4% DSS	0 ± 0	0 ± 0	0 ± 0	0 ± 0
AAT (125 mg/kg/day)	0 ± 0	0 ± 0	0 ± 0	0 ± 0
AAT (125 mg/kg/day) + 4% DSS	0.9 ± 0.6***	3.8 ± 1.2***	4.1 ± 1.7***	7.9 ± 2.0***
DMBA (25 mg/kg/day)	0 ± 0	0 ± 0	0 ± 0	0 ± 0
DMBA (25 mg/kg/day) + 4% DSS	1.0 ± 1.1**	1.5 ± 2.0*	1.5 ± 1.6**	3.0 ± 1.9***
ENU (11 mg/kg/day)	0 ± 0	0 ± 0	0 ± 0	0 ± 0
ENU (11 mg/kg/day) + 4% DSS	1.6 ± 0.5***	4.9 ± 1.9***	3.7 ± 1.5***	8.6 ± 1.1***

Multiplicity indicates the number of dysplasias, adenomas, or adenocarcinomas per mouse, mean ± standard deviation

CMNC; colon-mutagenic non-carcinogen

^*a*^: adenoma + adenocarcinoma

^*b*^: water for injection for 7 mice and salad oil for 6 mice

*, **, ***; significantly different from the DSS alone group at *P* < 0.1, 0.05, 0.01 in the Welch’s *t*-test

### Immunohistochemistry of β-catenin

The expression of the β-catenin protein in adenocarcinomas is shown in Fig. 5. In adenocarcinoma cells, whose formation was induced by AAT/DSS, DMBA/DSS, or ENU/DSS, β-catenin was translocated predominantly to both the nucleus and cytoplasm or only cytoplasm from cell membrane, where it is exclusively expressed in normal colon epithelial cells.

## Discussion

We previously reported that in mice administered BP, a CMNC, the formation of colon tumors was rapidly induced after DSS treatment [23, 24]. In the present study, we clearly showed that three more CMNCs (AAT, DMBA, and ENU) can rapidly induce the formation of colon tumors at a high multiplicity/incidence.

All tumors whose formation was induced by the three CMNC/DSS were histologically diagnosed as tubular adenomas or well- to moderately differentiated adenocarcinomas. These tumors predominantly appeared in the middle and distal parts of the colon, coinciding with the location of DSS-induced colitis. These findings were consistent with those reported for DSS in combination with BP or CMCs such as AOM, DMH, and PhIP [4, 7–12]. In adenocarcinoma cells, whose formation was induced by CMC in combination with or without DSS, or BP plus DSS, β-catenin was expressed in both the nucleus and cytoplasm or cytoplasm [4, 7, 8, 10, 24]. Such translocation and accumulation of β-catenin in the nucleus from cell membrane has been shown to be closely associated with the development or progression of colon tumors through mutations in the β-catenin or *APC* genes or activation of the Wnt/β-catenin signaling pathway [4, 40–43].

MeIQx, a heterocyclic amine found in well-cooked meat, is mutagenic but not carcinogenic in the colon of mice [44]. One study reported that MeIQx is carcinogenic in the mouse colon after DSS treatment [12]. However, the carcinogenicity was weak in that study (for adenomas plus adenocarcinomas, the incidence was 22% and multiplicity was 0.30 ± 0.61), and another study reported that MeIQx was not carcinogenic after DSS treatment [18]. Thus, the effect of DSS treatment on MeIQx-induced carcinogenicity is not clear and may be marginal. Another heterocyclic amine, IQ, was carcinogenic at a low incidence/multiplicity (for adenomas plus adenocarcinomas, the incidence was 14% and multiplicity was 0.34 ± 0.72) in mice after DSS treatment [12], although it is not reported to be a colonic carcinogen in itself [44]. IQ has been reported to show mutagenicity in the cecum, but not in the colon, at 20 mg/kg for 5 days [45]. In that study, only one dose was tested.

In rodent chemical-induced colon cancer models, CMCs, particularly AOM, are commonly used in combination with DSS. Our present and previous studies [23, 24] provide evidence showing that CMNCs can also induce colonic tumors after posttreatment with DSS, and that colonic mutagens can induce colonic tumors in the presence of DSS-induced colitis whether they are colonic carcinogens or not. To induce colonic cancer, CMNCs may play a key role in the induction of gene mutations responsible for tumorigenesis via their characteristic mutational spectrum.

**Fig. 6:**
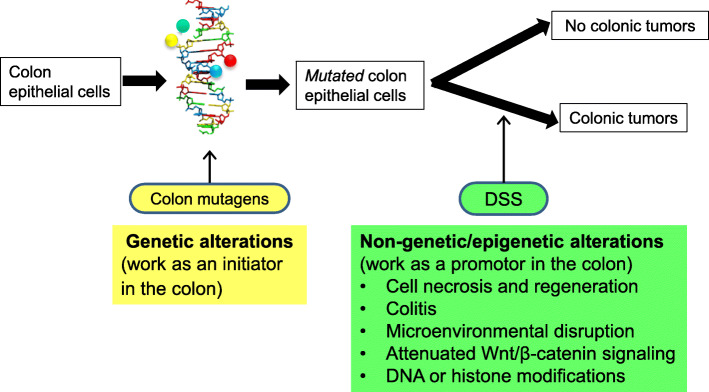
Hypothesis for possible mechanisms underlying the induction of colonic tumor formation after combined treatment with colonic mutagens and DSS. Colon epithelial cells are mutated by colonic mutagens, probably in stem or progenitor cells. Mutated epithelial cells develop tumors via non-genotoxic effects when mice are treated with DSS. When DSS is not treated, mutated epithelial cells do not develop tumors. Non-genotoxic effects of DSS include enhanced cell proliferation and inflammation in response to cell injury or microenvironment disruption, and thereby alterations in signal transduction or epigenetics. These effects are affected by intestinal bacteria. Colonic mutagens are not necessarily colonic carcinogens

Figure 6 shows our hypothesis for possible mechanisms of the induction of colonic tumors in the mouse colitis-associated model in combination with a colonic mutagen/carcinogen. Colon epithelial cells are mutated by colonic mutagens, resulting in the generation of mutated epithelial cells (probably stem cells or progenitor cells, because cell turnover of colon epithelial cells is very fast, i.e., 2–3 days [32, 33]). Mutated epithelial cells develop tumors via non-genetic effects when mice are treated with DSS, a colitis inducer and a potent promoter of carcinogenesis. In the absence of DSS treatment, mutated epithelial cells do not develop tumors. Non-genetic effects include cell regeneration accompanied by cell necrosis and inflammation in response to cell injury or microenvironment disruption, and thereby alterations in signal transduction [40–42, 46] or epigenetics (DNA methylation or histone modification) [47, 48]. These effects are affected by intestinal bacteria [49, 50]. Many studies have shown that CMCs rapidly induce colon cancer after treatment with DSS, and that colitis (inflammation) caused by DSS contributes to the rapid progression of colon carcinogenesis [4–6]. Therefore, inflammation may be a requisite factor for colon carcinogenesis and one of the discriminating factors of colon carcinogens from CMNCs. However, further studies are required for verification.

The colon becomes the site of tumor induction when juvenile *APC*^*Min/*+^ mice (carrying a mutation in the *Apc* gene to develop multiple intestinal adenomas) or even *APC*^+/+^ mice (wild-type at the *Apc* locus) are intraperitoneally treated with ENU, although the ENU does not induce colonic tumors when adult mice, generally used in toxicity studies, are orally treated [34, 35, 51]. In both strains of mice, the incidence of intestinal tumors age-relatedly reduces during the periods from 5 to 14 days to 30–35 days of age at ENU treatment, with tumors being induced at a very low incidence at 30–35 days of age [51]. The microenvironment of intestinal crypts dramatically changes from infancy to early juvenile; when mice are born, crypts are predominantly polyclonal, but around two to 3 weeks of age, they become monoclonal. This phenomenon, known as crypt purification or age-related differences in the DNA repair system, may decrease the number of cells initiated by ENU [51, 52].

Colon tumors are induced when adult *APC*^*Min/*+^ mice are treated with ENU [52]. This finding supports a canonical mechanism of colon tumorigenesis: the induction of mutations in the dominant tumor-suppressor gene. In contrast, our study showed that DSS post-treatment provides the same output of tumor induction as mutation induction in tumor-related genes in tumorigenesis.

Since the present study with the three colonic mutagens was performed under the same experimental conditions, the potency of their carcinogenicity in this model may be correlated and can be compared. The most powerful initiation activity was estimated to be that of ENU (0.33), followed by that of DMBA (0.060) and AAT (0.033), by calculating the multiplicity of adenocarcinoma divided by the dose (mg/kg/day).

The purpose of the present study was to provide evidence on the hypothesis that CMNCs act as initiators of carcinogenesis in a DSS-induced colitis model. Our study may help better understand the effects of environmental mutagens on inflammation-related cancer. Increasing attention has been paid to the involvement of inflammation in the initiation, promotion, and progression of tumors [53–56]. Patients with inflammatory bowel disease (IBD) are at a higher risk of developing colorectal cancer. The cumulative incidence of colorectal cancer in patients with IBD ranges from 7.6 to 18.4%, at 30 years post-diagnosis [5, 57]. A review paper by Rawla et al. [58] reported that patients with chronic IBD have a two-fold higher risk of developing colorectal cancer, and that ulcerative colitis increases the risk of colorectal cancer by 2.4 times. Our study suggests that colonic mutagens impose an increased risk of colon cancer in patients with IBD compared with healthy individuals.

## Conclusion

In our previous study, BP of a CMNC rapidly induced the formation of colonic tumors in mice after DSS treatment. In the present study, we clearly showed further evidence that the other three CMNCs (AAT, DMBA, and ENU) also rapidly induced the formation of colonic tumors in mice after DSS treatment. These findings indicate that colonic mutagens can cause colonic tumors in the presence of colitis due to DSS, regardless of whether they are carcinogenic in the colon.

## Data Availability

Data are available upon request. Material availability is not applicable.

## Abbreviations

AAT: *o*-Aminoazotoluene
AOM: Azoxymethane
BP: Benzo[*a*]pyrene
CMC: Colon-mutagenic carcinogen
CMNC: Colon-mutagenic non-carcinogen
DSS: Dextran sulfate sodium
DMBA: 7,12-Dimethylbenz[*a*]anthracene
DMH: 1,2-Dimethylhydrazine
ENU: *N*-Ethyl-*N*-nitrosourea
PhIP: 2-Amino-1-methyl-6-phenylimidazo[4,5-*b*]pyridine
